# Evaluation of Indian Prediction Models for Lung Function Parameters: A Statistical Approach

**DOI:** 10.5334/aogh.2397

**Published:** 2019-03-01

**Authors:** Ritul Kamal, Sheela Misra

**Affiliations:** 1 Department of Statistics, University of Lucknow, Lucknow, IN

## Abstract

**Background:**

Interpretation of lung function test parameters is usually based on comparisons of data with reference (predicted) values based on healthy subjects. Predicted values are obtained from studies of “normal” or “healthy” subjects with similar anthropometric and ethnic characteristics. Regression models are generally used to obtain the reference values from measurements observed in a representative sample of healthy subjects.

**Objectives:**

The study aims to carry out a statistical evaluation of the Indian prediction models of lung function parameters and critically evaluate the reference values for the same in an Indian context.

**Methods:**

The screening and inclusion of the articles for the study was done using the Preferred Reporting Items for Systematic Reviews and Meta-Analysis (PRISMA) guidelines. Evaluation of the prediction models has been done with respect to modeling approach, regression diagnostics and methodology protocol. The suitability of the models has also been evaluated using a checklist comprising of 8 criteria developed using the American Thoracic Society (ATS) guidelines.

**Results:**

Using the PRISMA guidelines 32 articles with a total sample size of 25,289 subjects were included in the final synthesis. Multiple linear regression models were used in 27 articles, with one additionally using weighted least squares technique and 4 using step-wise regression method. Regression diagnostics as per the ATS guidelines were performed and reported by 22 articles. The prediction models were traditionally developed using ordinary least squares method (OLS) without examining the homoskedasticity of residuals. The quality assessment using the checklist developed revealed that only 5 articles satisfied more than 7 out of 8 criteria, and a further 8 articles satisfied less than 3 criteria of suitability of prediction models.

**Conclusions:**

Indian prediction models for lung function models are traditionally based on linear regression models, however with more advancement in computational power for sophisticated statistical techniques, more robust prediction models are required in the Indian context.

## Introduction

Lung function tests play a vital role in diagnosing respiratory diseases such as asthma or chronic obstructive pulmonary disease (COPD), assessing disease severity and monitoring treatment responses [1,2,3].

Lung function capacity increases with age in childhood (due to growth and maturation) and declines with age in adulthood (due to loss of elastic recoil). It also depends on height as a proxy for chest size [4]. Lung function parameters are largely affected by various demographic, environmental, genetic and socioeconomic factors [5]. Pulmonary parameters, unlike other laboratory measurements, do not have a predefined “normal value” that is universally applicable to all individuals in a population. This variation in predicted value for lung function for each individual is due to difference in gender, race, thoracic cage and physical characteristics like age, weight and height. The predicted values for pulmonary function parameters are developed using regression equations. The data collected from healthy and non-smoking individuals of comparable demographic and socioeconomic status will be utilized for the regression equation [6]. Besides technical factors related to the procedures and equipment used, biological and environmental factors also contribute to the variations in the data. Racial, ethnic and anthropometric factors also contribute to the variation in the pulmonary parameters [7]. In a demographically and geographically diverse country like India, a lot of regional variations exist. The use of north Indian equations for south Indian individual may lead to substantial misclassification of the abnormality [7]. It therefore becomes imperative that relevant predictions equations are used while interpreting the pulmonary parameters [7,8].

Lung function parameters are generally found to be close to Gaussian distribution in the middle-age groups, but not for extreme values. The distributions of flow measurements and ratio measurements in lung function values are generally not symmetric [9]. Symmetric distributions about mean in these cases are obtained by either transformation or age stratification [9]. Ideally reference values for lung function parameters must not only include the prediction equations but also the means of defining their lower limits, which may be estimated using the regression models [1].

Prediction models using regression equations provide an efficient and economical method for describing the expected values of pulmonary parameters as a function of sex, height and age [1]. Regression models are based on the fundamental assumption that pulmonary function varies in a symmetric fashion about the mean value and the variance around the mean value remains constant from one observation to another [1]. Linear regression is the most commonly used model in Indian scenario to describe pulmonary function data in adults, however these models do not provide precise estimates around the tail of the distribution of data.

Evaluation of how well the regression model fits the data is done using regression diagnostic techniques. Predictive model fitting is considered as incomplete without running the regression diagnostics [10,11,12]. These diagnostic techniques are used to examine the fundamental assumptions of regression and also to assess the accuracy of the estimation for a multiple regression analysis model [13,14,15]. Regression diagnostics are used to check the validity of the fundamental assumptions of regression analysis, failing which the generalizability of the models becomes restricted.

The relationship between body size and lung function is of complex nature, especially during periods of rapid growth in human body [16,17,18,19], which results in the fact the traditional and most commonly used regression equations are insufficient for prediction modeling of lung function parameters. More recently there have been many advances in computational power and statistical software that allow for more sophisticated statistical methods to be applied with greater ease [19]. The increased flexibility allows for the complexities of these relationships to be quantified more accurately to reflect biologically and clinically plausible prediction models of lung function with age and height using a smoothly changing model [20].

The present study has been undertaken with the aim to carry out a statistical evaluation of the Indian prediction models of lung function parameters and to critically evaluate the reference values for the same in Indian context. The study will evaluate the statistical approach of prediction modeling, the parameters of regression diagnostics reported and the measurement of lung function parameters. The study will contribute to identifying the limitations and gap areas of the present Indian prediction models and identify further avenues of research in improving the methodology and application of prediction models in the Indian context.

## Methods

We searched the publications relating to Indian prediction equations for lung function parameters listed in the electronic database PubMed (source: https://www.ncbi.nlm.nih.gov/pubmed/) and Google Scholar (source: http://www.scholar.google.co.in) till July 1, 2018, using the following text and key words in combination: “Prediction equation,” “Pulmonary,” “Lung function,” “Prediction model,” “Regression,” “Spirometry” and “India.”

The included articles were screened and crosschecked independently by authors for relevance and suitability. The percentage of agreement between the authors on the quality of the articles ranged between 90–100%. All the disagreements were resolved by consensus among the authors. The references from the selected publications were also screened, and relevant articles were included in the analysis.

The search was limited only to articles in English. The search was limited to PubMed and Google Scholar due to the non-accessibility of Medline and Embase. The titles of the articles were first screened for possible relevance and exclusion. All the remaining articles were then considered as relevant for potential screening. In the case of articles where the full texts were not available, efforts were made to obtain the full texts by contacting the corresponding authors and journals. The articles received after that communication were subsequently screened for possible inclusion.

Identification, screening, eligibility, inclusion of articles and meta-analysis for the study follows Preferred Reporting Items for Systematic Reviews and Meta-Analysis (PRISMA) [21]. The systematic review protocol for PRISMA was based on the information available at http://www.prisma-statement.org/statement.htm.

The information abstracted from each of the selected articles included: author name, publication year, sample size, age group, gender, regression model, separate model for male and females, adjusted for smoking, reported regression diagnostic method, lung function parameters studied, instrument used and number of citations. The data for the citations of each article was also reviewed from Google Scholar (source: http://www.scholar.google.co.in).

The evaluation of the quality and suitability of the lung function prediction models was done using a checklist prepared using the recommendations of the American Thoracic Society [1] (Table 1). A checklist comprising of 8 criteria was prepared, and the quality of each prediction model was evaluated on the basis of those criteria. The overall score for each article was calculated in percentage. The quality scores were then plotted against the number of citations for each article to assess the use of the prediction models for biological and clinical interpretation of lung function parameters.

**Table 1:** Quality checklist for assessment of the suitability of prediction models as per the ATS guidelines.

S No	Assessment criteria

1	Use of acceptable methods and equipment for measurement of lung function parameters
2	Adequately defined sample size for prediction models
3	Adequately described statistical methodology protocol for prediction equation generation
4	Reporting parameters of regression diagnostics
5	Validation of prediction models on independent study samples
6	Inclusion of age and height as independent predictor variables for lung function parameters
7	Separate prediction models for male and female subjects
8	Reporting lower limit of normal values or information regarding calculation of the same

## Results

**Figure 1:**
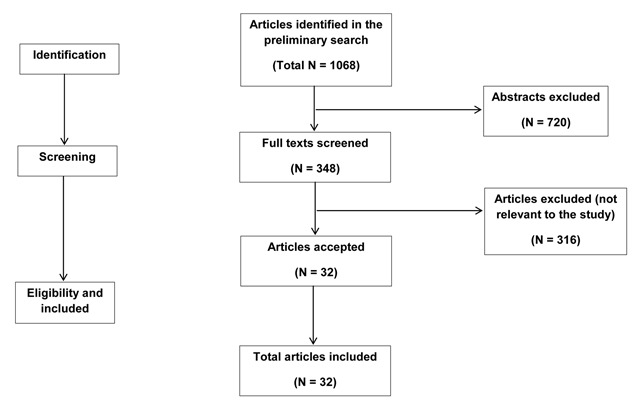
Flowchart for inclusion of articles in the study.

During the initial search using keywords, 1,068 titles related to keywords were retrieved. The abstracts of all the articles selected after initial search were evaluated for possible inclusion. Out of these, 348 articles were considered relevant, and the full texts of these were retrieved for detailed examination and scrutiny. Out of the 348 articles, 316 were subsequently excluded due to not being relevant to the analysis, and so 32 articles were included in the final study. The detailed procedure for the inclusion of articles is presented in Figure 1.

The characteristics of the included articles (author name, publication year, sample size, age group, gender, regression model, separate model for male and females, regression diagnostic reported, pulmonary function parameters studied, instrument used and number of citations) in the final synthesis are shown in Table 2. Thirty-two articles on prediction equations for various lung function parameters among subjects in India with a total sample size of 25,289 were identified after screening and included in the study.

**Table 2:** Characteristics of the articles for Indian prediction models included in the analysis.

S No	Author	Sample size	Age group	Gender	Regression model	Separate model	Regression diagnostics	Pulmonary parameters	Instrument used	Detailed method	Quality Score	Citations

1	Parmar et al. 1977	595	6–16 years	Boys and Girls	Linear regression model	Yes	No coefficient reported	PEFR	Wright Peak Flow meter	No	37.5	37
2	Singh & Peri et al. 1978	663	4–16 years	Boys and Girls	Linear regression model	Yes	R^2^, SEE reported	PEFR	Wright Peak Flow meter	No	37.5	17
3	Singh et al. 1979	851	17–70 years	Males and Females	Linear regression model	Yes	R, SEE reported	PEFR	Peak Flow meter	No	62.5	34
4	Malik et al. 1981	605	5–16 years	Boys and Girls	Linear regression model	Yes	R^2^, SEE reported	PEFR	Wright Peak Flow meter	No	50	30
5	Gupta et al. 1982	1427	3–22+ years	Males and Females	Linear, Interaction, General, Proportional, Multiplicative, Polynomial	Yes	RSD reported, homogeneity of variance tested	PEFR	Wright Peak Flow meter	No	75	31
6	Aundhakar, et al. 1985	515	6–15 years	Boys and Girls	Linear regression model	Yes	R^2^ reported	PEFR	Wright Peak Flow meter	No	37.5	9
7	Udwadia et al. 1987	760	<30 years and> 30years	Males and Females	Multiple linear regression model	Yes	R, R^2^ and SD around the regression line reported (S_y.x_).	FEV_1_, FVC, FEV_1_/FVC, PEF, FEF25–75%, Vmax50%, Vmax75%	Computerized spirometer	No	87.5	37
8	Chatterjee et al. 1988	334	20–60 years	Males	Multiple linear regression model	No	R, R2 and SEE	FEV_1_, FVC, FEV_1_/FVC, PEF, FEF25–75%, Vmax50%, Vmax75%	Wright Peak Flow meter	Yes	75	18
9	Vijayan et al. 1990	247	15–40 years	Males and Females	Linear regression model	Yes	R^2^, SEE reported	FVC, FEV, FRC, TLC, VA	Spirometer	Yes	87.5	80
10	Dikshit et al. 1991	127	55–85 years	Males	Linear regression model	No	R coefficient reported.	PEFR	Wright Peak Flow meter	No	25	16
11	Mohan Rao et al. 1992	96	15–40 years	Males and Females	Multiple linear regression model	Yes	R, R^2^ and SEE	FVC, FEV_1_, FEF25–75%, PEFR	Wright Peak Flow meter	No	50	10
12	Ray et al. 1993	2000	10–59 years	Males and Females	Multiple linear regression model	Yes	R^2^ reported	PEFR	Wright Peak Flow meter	Yes	87.5	23
13	Swaminathan and Venkatesh et al. 1993	345	4–15 years	Boys and Girls	Linear regression model	Yes	No coefficient reported	PEFR	Wright Peak Flow meter	No	50	51
14	Chowgule, Shetye & Parmar et al. 1995	632	6–15 years	Boys and Girls	Linear regression model	Yes	R^2^ reported	PEFR	Wright Peak Flow meter	No	50	66
15	Sharma et al. 1997	410	10–15 years	Boys and Girls	Linear regression model	Yes	R^2^ reported	PEFR	Wright Peak Flow meter	No	62.5	22
16	Pande et al. 1997	1257	6–17 years	Boys and Girls	Linear regression model	Yes	R^2^, SEE reported	PEFR	Wright Peak Flow meter	No	62.5	21
17	Rajkapoor et al. 1997	186	6–13 years	Boys and Girls	Linear regression model	Yes	No coefficient reported	PEFR	Wright Peak Flow meter	No	50	17
18	Harikumaran et al. 1997	109	5–16 years	Boys	Multiple linear regression model	No	No coefficient reported	VC, IVC, FVC, FEV1, PEF, FEF, PIF, FMFT, MVV	Computerized spirometer	No	25	28
19	Vijayan et al. 2000	469	7–19 years	Boys and Girls	Linear regression model	Yes	No coefficient reported	PEFR	Wright Peak Flow meter	No	50	66
20	Verma SS et al. 2000	173	8–13 years	Boys and Girls	Linear regression model	No	R^2^, SEE reported	PEFR	Wright Peak Flow meter	No	50	7
21	Sitarama Raju et al. 2003	1555	5–15 years	Boys	Linear, quadratic, cubic or logarithmic	No	R, R^2^ and SE of estimate reported.	FVC, FEV_1_, PEFR, FEV_1_/FVC	Wright Peak Flow meter	Yes	50	52
22	Sitaram Raju et al. 2004	1038	5–15 years	Girls	Step-wise linear regression model	No	R^2^, SEE reported	FEV_1_, FVC, PEFR, FEV_1_/FVC	Wright Peak Flow meter	Yes	37.5	1
23	Dikshit et al. 2005	Not mentioned	<20 years to> 60 years	Males and Females	Linear regression model	Yes	R, SEE reported	PEFR		No	75	50
24	Raju et al. 2005	2616	5–15 years	Boys and Girls	Step-wise linear regression model	Yes	R, F and SEE reported	FEV_1_, FVC, PEFR	Wright Peak Flow meter and Spirometer	Yes	75	39
25	Prasad et al. 2005	897	10–60 years	Males and Females	Step-wise linear regression model	Yes	R, R^2^ and RSD reported	PEFR	Wright Peak Flow meter	Yes	75	59
26	Mathur et al. 2007	137	20–68 years	Males	Weighted least squares method	No	R^2^, RSD reported	PEFR	Wright Peak Flow meter	Yes	62.5	29
27	Saleem et al. 2011	3080	18–65 years	Males and Females	Multiple linear regression model	Yes	R^2^, SEE reported	FVC, FEV1, PEFR, FEF25–75	Spirometer	No	75	2
28	Jacob et al. 2013	1165	5–17 years	Boys and Girls	Linear regression model	Yes	R, R^2^ reported	PEFR	Not specified	No	62.5	7
29	Chhabra et al. 2014	685	18–60 years	Males and Females	Multiple linear regression procedure. Linear and non-linear models	Yes	R^2^, Adjusted R^2^ and SEE reported	FVC, FEV_1_, PEFR, FEF_25–75_, FEF_50_, FEF_75_, FEV_1_/FVC	Spirometer	Yes	100	21
30	Shivkumar et al. 2014	91	10–15 years	Boys	Step-wise linear regression model	No	Adjusted R^2^ reported	FVC, FEV_1_, FEV_1_/FVC,MEF25, MEF50, MEF75, MMEF, PEF	Spirometer	No	14.28	0
31	Dasgupta et al. 2015	706	15–69 years	Males and Females	Multiple linear regression model	Yes	R^2^, SEE reported	FEV1, FVC	Spirometer	Yes	100	1
32	Pramanik et al. 2015	1518	10–18 years	Boys	Linear regression model	No	No coefficient reported	FVC, FEV_1_, PEFR, FEF25–75%	Spirometer	No	37.5	2

Among the 32 articles analyzed, 7 articles included only male subjects, 1 article included only female subjects and the remaining 24 articles included both male and female subjects for a prediction model for lung functions. Out of the 32 articles included in the analysis, 23 reported separate prediction models for male and female subjects, whereas the rest of the articles reported only one model for prediction for either gender. In terms of the spirometer used for measuring the lung function parameters, 22 articles reported using the Wright Peak Flow meter, 8 articles reported using Spirometer and no specific information regarding the instrument was mentioned in two articles.

Linear regression models were used for prediction of pulmonary parameters in 27 articles. In addition to linear models, non-linear models developed by Chabbra et al. [6], Raju et al. [22] and Gupta et al. [23] were also used in different studies. Step-wise linear regression models were used for prediction models for 4 articles, and Mathur et al. [24] used the weighted least squares approach for modeling the pulmonary parameters.

Out of the 32 articles included, 26 articles reported regression diagnostics, and the other 6 articles did not report any regression diagnostic coefficient. Prediction models in 22 articles provided correlation coefficients and coefficient of determination for the equations developed, and 18 articles additionally provided the standard error of the estimate (SEE).

Only 10 articles defined the detailed methodology protocol of the prediction equations, which in statistical terms provides a basis for better evaluation of the prediction model. The minimum sample size required for multivariate regression analysis of the lung function parameters is 150 for validation of prediction models [25], however 6 articles included in the analysis did not satisfy the sample size criteria for prediction of lung function parameters. All the included articles used acceptable methods and equipments for measurement of lung function parameters. Ten out of 32 articles provided independent validation of the prediction models. Four out of 32 articles selected mentioned about the lower limit of normal values or presented information from which the lower limit of normal values may be obtained. The ATS guidelines for the suitability of prediction models suggest that prediction models for lung function parameters include age and height as independent predictor variables. However, 8 articles did not include both age and height as variables in the prediction models.

In 29 articles, the regression coefficients for prediction variables of lung function parameters were calculated using ordinary least square (OLS) approach without examining the homoskedasticity of residuals. The heteroskedasticity of residuals was examined by Prasad et al. [14] and Mathur et al. [24], as reported in the articles.

In regression model, each data point must provide equally precise information about the deterministic part of the total variation (i.e., the standard deviation of the term must be constant over all values of the predictor variables). However, this assumption does not always hold well in case of OLS model. In such circumstances, precise estimates of regression coefficients were obtained using two approaches viz. transforming the data or using weights. The evaluation of the prediction models revealed that the prediction models using transformed variables were presented in 6 articles, and additionally, Mathur et al. used weighted least squares method [24].

The total number of citations for the articles included in the analysis was 882. The number of citations ranged from no citations for Shivkumar et al. [26] to 80 for Vijayan et al. [27].

The quality assessment of the prediction models using the checklist developed based on the ATS guidelines revealed that only 2 articles satisfied all the criteria of suitability of prediction models and 3 articles satisfied seven out the eight criteria of suitability. In total, 8 articles satisfied less than three criteria of suitability of prediction model.

**Figure 2:**
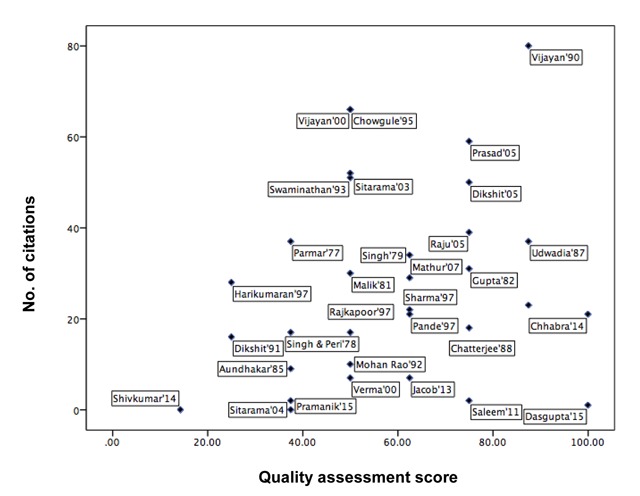
Scatter plot of the relationship between the quality assessment score and number of citations of the selected articles.

The quality assessment score for the prediction models was plotted as percentage value against the number of citations of the respective article. Reference values obtained from these prediction models are used for the biological and clinical interpretation lung function status. The reference values must ideally come from prediction models that satisfied all the criteria for suitability as recommended by the ATS. However, many articles that scored high on the quality assessment had low citations as compared to articles with lower scores. The correlation coefficient between the quality score and number of citations was 0.22 (Figure 2), indicating a weak linear relationship between quality assessment score and number of citations.

## Discussion

### Evaluation of statistical approaches on development of prediction model for lung functions in the Indian population

The American Thoracic Society (ATS) has suggested various statistical considerations for prediction of lung parameters. These considerations include separate equations for male and female subjects, as well as separate equations based on ethnicity [9]. Linear regression equations perform adequately for adults, however they have a tendency of overprediction in young adults and underprediction in the elderly. It is also stated that prediction equations must come from studies that present lower limits of normal or provide information about calculation of the same. Care must be taken while extrapolating the reference equations and should correlate and validate with clinical findings [1]. Indian prediction equations are conventionally calculated using linear regression models without providing details about the statistical methodology of the prediction modeling and the information about the lower limit of normal. Additionally, the regression diagnostics techniques, which are essential for model building and examination of the fundamental assumptions of regression to assess the accuracy of prediction, are often not reported in the articles.

In the present study, statistical evaluation of parameters of regression diagnostics reported by the prediction equations was undertaken on the basis of coefficient of determination (R^2^) and the reported SEE of the constants and regression coefficients. The goodness of fit for the regression model is generally reported using the coefficient of determination (R^2^) and the standard error of the estimate (SEE). The proportion of the variability in the observed data explained by the predictor variables is given by the R^2^ value, and SEE is the average SD of data around the fitted regression line. As the differences between the predicted and observed values of lung function parameters in the reference population diminishes, the SEE value decreases, and correspondingly R^2^ increases [1]. Because these statistics reflect the average characteristics of the regression models, the R^2^ and SEE values may be able to define the ability of the prediction model to describe the tails of the distributions or the lower limit of “normal” value, and hence they are insufficient criteria to chose the best prediction model to clinically evaluate a population [1].

The statistical considerations for lung function prediction equations as suggested by the ATS were also evaluated in the present study. The evaluation was carried out on the parameters for reporting of detailed statistical methodology of regression models, availability of lower limit of normal vales or information for the calculation of the same and accompanying validation data on an independent data set for testing the validity of the prediction models.

### Gap areas of the present Indian prediction equations

Conventionally, in the Indian context the lung function pulmonary parameters have been modeled using traditional linear regression models, with the assumption of homoscedasticity and normality of residuals [4]. Linear regression models are based on four basic assumptions: (1) a linear relationship, (2) constant variability of values around the mean, (3) a normally distributed outcome variable and (4) combined effect of covariates is additive. However, in the case of pulmonary parameters, these assumptions are rarely met [28]. The prediction models are fitted, using the OLS approach, without examining the homoskedasticity of residuals with respect to predictor variables, which is an essential part of statistical model building [14,29,30]. The residuals often are heteroskedastic in nature (i.e., the standard deviation of the error term is not generally constant over all values of the predictor variables). Thus the assumption of constant variability around the mean values does not hold well in the case of lung function parameters. The linear prediction models using the OLS approach have very limited extrapolation properties over a large range of values and are also highly sensitive to outlying observations. The presence of only a few outliers can skew the results of the OLS model; hence, the model diagnostics and validation are very critical [30].

The step-wise regression model used in some articles provides more power and information than OLS procedure. It allows the handling of numerous predictor variables, fine-tuning the model for choosing the optimum predictor variables [30]. However, the procedure has its own disadvantages because collinearity is a major issue. The R^2^ and adjusted R^2^ values are too high. Additionally, the predicted values and confidence interval of the estimates are often too narrow [30].

### Future avenues for research in improving the methodology and application of prediction models in the Indian context

Quantile regression, in comparison to linear regression, is a more adequate method to calculate reference ranges because it makes no distributional assumption and allows an independent estimation of conditional quantile functions resulting in reference limits, which are independent of global parameters like the standard deviation. Furthermore, the quantile regression shows a high robustness to outlier observations [31,32,33]. Another possible alternative to the linear regression models is the use of the LMS (lambda, mu, sigma) method. The LMS method is an extension of regression analysis that includes three components: (1) the median (mu), which represents how the outcome variable changes with an explanatory variable (e.g., height or age); (2) the coefficient of variation (sigma), which models the spread of values around the mean and adjusts for any non-uniform dispersion; and (3) the skewness (lambda), which models the departure of the variables from normality using a Box-Cox transformation. The method is widely used to construct growth reference charts [19].

The more recent generalized additive modelling of location, scale and shape technique (GAMLSS) provides an extension to the LMS method [34]. The flexibility of GAMLSS method allows an extended class of models to be fitted, where the distribution of the lung function parameters depends not only on age, including the child–adult transition, but also on one or more measures of size [19].

In recent years, many advances have occurred in development of lung function prediction equations, such as development of standardized measurement protocols across all age groups, including those for preschool children [8,35,36]; more robust and appropriate statistical techniques for developing prediction models [28,37,38]; and establishment of various international collaborative networks with open access to Spirometry and other related data in healthy test subjects [20].

## Conclusions

India has the second largest population in the world, having 18% share of the global population. However, India has a disproportionately high percentage (32%) of the global DALYs from chronic respiratory diseases [39]. To mitigate the growing burden of the respiratory diseases, suitable strategies need to be framed, which require updated and appropriate reference values. To achieve the same, more efforts are needed to produce robust prediction models for lung function parameters in the Indian context. Age, gender and ethnicity specific prediction models along the independent validation data and lower limit of normal values are required in the Indian context. The lack of data to validate the prediction models from the articles included in the analysis remains a limitation of the study.
